# Management of Advanced Laryngeal Cancer

**DOI:** 10.5041/RMMJ.10149

**Published:** 2014-04-28

**Authors:** Patrick Sheahan

**Affiliations:** South Infirmary Victoria University Hospital, Cork, Ireland

**Keywords:** Chemoradiotherapy, laryngeal neoplasms, laryngectomy, larynx, radiotherapy, squamous carcinoma

## Abstract

Squamous cell carcinoma of the larynx continues to be the commonest head and neck cancer in many Western countries. The larynx plays a key role for many essential functions, including breathing, voice production, airway protection, and swallowing. The goals of laryngeal cancer treatment are thus to provide best possible oncologic control, while optimizing functional outcomes.

In recent decades, the treatment paradigm for advanced laryngeal cancer has shifted from one of primary surgery (total laryngectomy) as gold standard, toward non-surgical organ-preserving treatment using radiotherapy or chemoradiotherapy. However, concerns have emerged regarding functional outcomes after chemoradiotherapy, as well as possible decreased overall survival in patients with laryngeal cancer.

The purpose of the present review is to review surgical and non-surgical options for treatment of advanced laryngeal cancer, as well as the evidence supporting each of these.

## INTRODUCTION

Squamous cell carcinoma (SCC) of the larynx continues to be the commonest cancer of the head and neck in many Western countries. Major risk factors include smoking^1^,^2^ and alcohol consumption.^1^–^3^ Other risk factors include asbestos exposure,^4^,^5^ industrial pollution,^6^ history of larynx cancer in a first-degree relative,^7^ and inadequate intake of anti-oxidant micronutrients found in fresh fruit and vegetables.^8^–^10^ Males are more commonly affected, and most patients are aged over 40 years. While many countries have recently reported a decline in overall number of cases of larynx cancer, it would appear that this decrease is mainly due to the decreased number of cases affecting males, with a stable or increasing number of cases affecting females.^11^ These changes in epidemiology of larynx cancer have been attributed to changes in smoking patterns.

The larynx has a key role in many essential functions, including speech production, swallowing, airway protection, and breathing. Disruption of any of these functions, by either the tumor or the treatment, may have devastating consequences for the patient. Therefore, besides achieving tumor control, the other major aim of laryngeal cancer treatment is to optimize functional outcomes. Although this is usually possible in early larynx cancers, preserving laryngeal function in the setting of advanced cancer while still offering the optimum oncological outcome can be a difficult challenge.

## DEFINITION OF ADVANCED LARYNGEAL CANCER

The term advanced laryngeal cancer generally denotes stage 3 or 4 laryngeal cancers according to the Union for International Cancer Control (UICC) / American Joint Committee on Cancer (AJCC) staging.^12^ Laryngeal cancers may attain this advanced stage classification by virtue of advanced T classification (T3 or T4), N classification (N1–3), or M classification (M1). It should be noted that this definition of advanced laryngeal cancer allows for the inclusion of cases with early T classification (T1/2), but meeting criteria as advanced stage on the basis of nodal disease. While nodal disease is well established as an adverse prognosticator in larynx cancer, it has been argued that inclusion of cases with early T classification in organ preservation trials may introduce bias in trials where the major end-points are local control and/or laryngeal preservation.

Laryngeal cancers attain T3 classification if they have vocal cord fixation, paraglottic space invasion, pre-epiglottic space invasion, postcricoid extension, or minor thyroid cartilage erosion. T4 classification is attained in tumors with cartilage destruction or extralaryngeal invasion.^12^ Accurate staging of larynx cancers demands careful clinical and radiological assessment. One of the challenges in staging these cancers is the subjectivity which may be involved in the defining criteria for T3 classification. Thus, vocal cord fixation is an important criterion for defining T3 classification and, when present, is generally accepted to have a significant adverse impact on likelihood of control with non-surgical treatment.^13^,^14^ However, vocal mobility may be difficult to assess in the presence of a bulky tumor obstructing visualization. Furthermore, differentiation between reduced movement (T2b) and vocal fixation (T3) can be difficult. The other defining criteria for T3 classification also involve a certain extra level of subjectivity and may depend on the type and quality of imaging performed and radiological interpretation. For example, minor erosion of the inner lamina of thyroid cartilage is notoriously difficult to diagnose with a high level of accuracy, yet the presence of this may upstage a small glottic cancer from T1 to T3. On the other hand, T3 tumors may include bulky tumors plastered along the whole inner lamina of thyroid cartilage, with many areas suspicious for erosion, but without any definite areas of gross cartilage destruction which would upstage the tumor to T4. It would seem very intuitive that the latter represents a much less favorable scenario than a smaller tumor with one focally equivocal area. Likewise, paraglottic or pre-epiglottic space involvement may include a spectrum from cases of very early involvement of these spaces diagnosed on the basis of subtle and possibly subjective radiological appearances, which is still easily amenable to transoral laser resection, to extensive and bulky involvement, which is not amenable to any form of conservation laryngeal surgery, and with decreased likelihood of local control with non-surgical treatment.

T4 tumors are subdivided into T4a or T4b, with T4b being defined as tumors with encasement of the common carotid artery, invasion of prevertebral fascia, or direct invasion of the superior mediastinum. The importance of the T4b classification is that such tumors are usually considered inoperable without leaving grossly positive margins, and thus such cases are generally considered not appropriate for primary surgical treatment.

## PRESENTATION

The majority of glottic cancers present at an early stage, due to the presence of hoarseness as an early symptom, while the poor lymphatic drainage of the glottis means that cervical metastases are rare with early primary tumors (<5%). Glottic cancers usually reach an advanced stage after involvement of the ventricle, with subsequent invasion of the paraglottic space and extension to the supraglottis. Vocal cord fixation is an ominous sign, which may arise from bulky involvement of the vocal cord and paraglottic space, or involvement of the cricoarytenoid joint. Destruction of thyroid cartilage and extralaryngeal extension is a late sign which upstages the tumor to T4 classification. It is possible that many advanced glottic cancers actually arise primarily within the laryngeal ventricle, which facilitates early spread to the paraglottic space and supraglottis. So-called transglottic cancers, involving both supraglottis and subglottis, appear to have a particularly unfavorable biology. However, even advanced glottic cancers have a relatively low incidence of cervical metastases (approximately 10%).

In contrast, supraglottic cancers may grow to a considerable size before causing symptoms, and, due to the rich lymphatic drainage, they commonly have nodal metastases at presentation. Thus, most supraglottic cancers present at an advanced stage, either due to local symptoms from a large tumor, or with a metastatic neck lump. Supraglottic cancers rarely show inferior extension below the level of the glottis. More problematic is spread to the vallecula and base of tongue, and extralaryngeal extension in the region of the thyrohyoid membrane. Nodal metastases are common, even in the presence of a clinically negative neck (30%–40%). Lymph nodes in levels 2A and 3 comprise the first echelon of drainage, and metastatic spread to both sides of the neck is commonly seen. Thus, treatment of early or advanced supraglottic cancer generally requires simultaneous addressing of both sides of the neck.

## TREATMENT

Definitive treatment options for advanced laryngeal cancer include surgery, radiotherapy, chemoradiotherapy, or a combination of these.

Surgical options may range from minimally invasive transoral laser or robotic surgical resection, to open partial laryngectomy, to total laryngectomy. However, for many cases of advanced larynx cancer, the only feasible option is total laryngectomy. In the past, this operation was considered to be the gold standard treatment for advanced laryngeal cancers.^15^ However, while it offers excellent local control, it is associated with significant functional and psychological sequelae.

More recently, there have been major changes in treatment paradigms for advanced laryngeal cancer. The result has been a major decrease in the number of patients treated with surgery alone, and a major increase in the number of patients treated with radiotherapy and chemoradiotherapy. The major driver for these changes has been the publication of clinical trials reporting high rates of larynx preservation after using chemoradiotherapy protocols to treat advanced laryngeal cancer.^14^,^16^ However, simultaneous with this shift in treatment paradigm, new concerns have emerged after the recent publication of data which would appear to show a reduction in larynx cancer survival over recent decades.^17^

An important factor which facilitates non-surgical treatment of advanced laryngeal cancer is the anatomy of the larynx and the impact of this on the pattern of post-radiotherapy recurrences. Thus, due to the anatomical constraints of the larynx, and the barriers to invasion provided by the laryngeal cartilages and membranes, when cancers which are originally confined to the larynx fail initial treatment with radiotherapy, the recurrent cancers also tend to remain confined to the larynx. Because of this, post-radiotherapy recurrences are usually amenable to surgical salvage by means of total laryngectomy with a reasonable expectation of disease control. This is in contrast to most other head and neck cancers, which are much less likely to be salvageable if they recur after initial non-surgical treatment.

### Conservation Laryngeal Surgery

Conservation surgery (transoral laser or robotic surgery, or open partial laryngectomy) is an excellent option for many patients with early (T1/2N0) larynx cancers, offering excellent oncologic control and functional outcomes.^18^–^20^ For advanced cancers, the role of conservation surgery is much more limited to cases which are either early T stage, but with concurrent cervical metastases, or select small-volume T3 cases.

One of the drawbacks with conservation surgery for advanced laryngeal cancer is the risk of greater functional deficit and higher risk of complications with more extensive resections. For example, resection of one arytenoid cartilage during supracricoid laryngectomy has been shown to lead to increased risk of aspiration pneumonia, longer time to decannulation of tracheostomy tube, and poorer voice.^21^–^25^ Thus, the functional advantages of conservation surgery over non-surgical treatment may be less clear-cut. Another concern is that, in patients with palpable neck disease, concurrent neck dissection will need to be undertaken with the surgery, and postoperative radiotherapy will in most cases be recommended to optimize regional control. The administration of postoperative radiotherapy may also adversely affect functional outcomes, although as long as the dose to the larynx is kept at 50 Gy, the adverse impact should be within acceptable limits.^26^,^27^ Finally, in the case of cancers undergoing open partial laryngectomy, patients will need to consent to proceeding to possible immediate total laryngectomy based on intraoperative findings and frozen sections. Total laryngectomy may also need to be considered in cases with positive margins at final histology. The risk of positive margins and possible need for total laryngectomy is more likely to be an issue for locally advanced primary tumors than for smaller primary tumors. However, given that many such cases are likely to be also amenable to treatment with radiotherapy or chemoradiotherapy with a reasonable expectation of good outcome, getting patients’ consent for an operation which may end up with total laryngectomy may be a “hard sell.”

Nevertheless, for well-selected cases of “intermediate-stage” laryngeal cancer, conservation laryngeal surgery effected either by transoral laser or open partial surgical techniques can offer excellent oncological and functional outcomes.^28^–^32^ Cases most suitable for a conservative surgical approach will be those staged T3 based on minor pre-epiglottic or paraglottic space invasion or minor inner lamina of thyroid cartilage erosion, without full restriction of vocal mobility (indicating absence of arytenoid fixation), in motivated patients with good performance status and pulmonary reserve.

### Non-Surgical Treatment

The Veterans Administration (VA) study in 1991 marked a major change in attitudes toward treatment of advanced laryngeal cancer.^14^ This was a randomized controlled trial comparing two treatment arms. Inclusion criteria were patients with stage 3 or 4 laryngeal cancer. The first arm underwent 2–3 cycles of induction chemotherapy, followed by definitive radiotherapy provided there was tumor response to chemotherapy. Non-responders underwent immediate total laryngectomy. The second arm underwent total laryngectomy with postoperative radiotherapy. Two-year survival was equal in both arms (68%); however, 36% of the non-surgical arm retained their larynx. Thus, this study was taken as evidence to support the use of primary chemoradiotherapy as treatment for advanced laryngeal cancer, on the basis that it offered patients an equal survival, but with a two-thirds likelihood of retaining their larynx.

The VA study was followed by a further landmark study, the Radiation Therapy Oncology Group (RTOG) 91-11 study published by Forastiere et al. in 2003.^16^ This comprised a three-arm randomized controlled trial on patients with stage 3/4 laryngeal cancer. The first arm consisted of induction chemotherapy followed by radiation; the second consisted of concurrent chemoradiotherapy; and the third consisted of radiotherapy alone. This study showed a superior locoregional control and laryngeal preservation rate in the concurrent chemoradiotherapy group, although there was no difference in overall survival and a higher incidence of severe toxicity in the concurrent chemoradiotherapy arm. This study was a major driver for primary chemoradiation to become the first-line treatment for most patients with advanced laryngeal cancer.

Both the VA study and the Forastiere study have been criticized on a number of grounds. One was the inclusion of some patients with early-stage primary tumors, but considered to have advanced laryngeal cancer on the basis of cervical metastatic disease. For example, nearly half of patients in both studies had mobile vocal cords. Given that the end-point of these trials was laryngeal preservation, this may have biased the results toward showing a better outcome from non-surgical treatment. Indeed, a French randomized controlled trial limited to patients with T3 primary tumors, which compared total laryngectomy to induction chemotherapy followed by radiotherapy in responders (or total laryngectomy in non-responders), demonstrated a significantly better survival in the group undergoing immediate surgery.^33^

Another criticism was the short follow-up, with only 2-year survival data reported in the original papers. In a recent update to the RTOG 91-11 study, 10-year survival data are reported. These results are very interesting insofar as while they confirm a superior laryngeal preservation rate and locoregional control for patients treated with concurrent versus induction chemotherapy, there was no significant difference in laryngectomy-free survival. Differences in overall survival were not significantly different; however, there was a trend toward a worse survival in the arm treated with concurrent chemoradiotherapy, which was attributable to an increased number of deaths which were apparently unrelated to the index cancer in the concurrent chemoradiotherapy group.^34^ These long-term findings might suggest that the increased incidence of toxicity in the concurrent chemoradiation group may be consequential in leading to increased mortality in the ensuing years.

The final criticism is that while these studies reported an impressive laryngeal preservation rate among patients treated non-surgically, little information was given regarding the function of the preserved larynx. In recent years, this has emerged as a major concern in patients treated with primary chemoradiotherapy. Secondary analyses of patients enrolled in clinical trials of chemoradiotherapy in head and neck cancer have reported severe late toxicity in 39%–43% of evaluable patients,^35^,^36^ with laryngopharyngeal primary site, older age, and advanced T stage being predictors for worse outcome.^35^ A systematic review of studies reporting on the incidence of pharyngo-esophageal stricture after radiotherapy reported an overall incidence of stricture of 7.6%, but rising to 16.7% in the intensity-modulated radiotherapy group (where most patients also received chemotherapy), and also being three times higher in prospective than retrospective studies,^37^ while rates of permanent gastrostomy tube use as high as one-third have been reported.^38^ In particular, for patients with dysfunctional larynges prior to treatment commencement, a dysfunctional larynx post treatment is to be expected.

Since the publication of the RTOG study, further studies have been performed investigating the role of TPF (taxane, cisplatin, and 5-fluorouracil) versus PF (cisplatin and 5-FU), as was used in the RTOG trial, for induction treatment. Pointreau et al. reported a better response rate to induction treatment (80% versus 59%), and better 3-year laryngeal preservation (70% versus 57.5%) with TPF induction versus PF induction followed by radiotherapy in patients with SCC of the larynx or hypopharynx. Differences in overall and disease-free survival were not significantly different.^39^ This was consistent with earlier findings from Posner et al. who found TPF induction followed by chemoradiotherapy to have superior survival in patients with head and neck cancer from all sites.^40^ These findings, along with the long-term findings of the RTOG 91-11 study, have led to a renewed interest in sequential chemoradiotherapy. However, the drawback of a more prolonged treatment regime may be reduced compliance, particularly among patients with poorer performance status. On the other hand, response to induction chemotherapy may be a very useful predictor of response to radiotherapy, and so may help select patients with very advanced tumors for definitive surgical versus non-surgical management.^41^,^42^

Thus it is clear that the major advantages of radiotherapy or chemoradiotherapy for treatment of advanced laryngeal cancer are avoidance of an operation and anatomic preservation of the larynx, with no definite compromise in overall survival.^14^,^43^,^44^ On the other hand, the disadvantages include a high incidence of severe acute toxicity, and a high incidence of long-term laryngeal functional problems, particularly in patients treated with concurrent chemoradiotherapy.^35^–^38^ There also appears to be a reduced likelihood of local control for patients with T4 tumors with gross cartilage destruction or extralaryngeal extension. Thus, consideration toward primary total laryngectomy should be given in these patients. Furthermore, among patients who develop local recurrence and require salvage laryngectomy, there is an increased incidence of pharyngocutaneous fistula and major complications in the post-radiotherapy setting.^45^

At most institutions, radiotherapy or chemoradiotherapy is the treatment of choice for most T3 laryngeal cancers. The decision to enhance the radiotherapy with chemotherapy will depend mainly on the patient’s general condition, medical co-morbidity, and ability to tolerate chemotherapy. Frail patients or patients with medical co-morbidity are best treated by radiotherapy alone; the possible benefit in local control by adding chemotherapy in such patients may be more than offset by the increased risk of local recurrence due to breaks in treatment caused by acute toxicity. For patients aged >70 years, the addition of chemotherapy has not been shown to offer any benefit over radiotherapy alone, while functional outcomes have been reported to be even worse. Another consideration may be whether there is likely to be a conservation surgical option in the event of treatment failure. Whereas conservation laryngeal surgery may be an option in some highly selected patients with recurrent laryngeal cancer after radiotherapy, this will almost never be feasible in the post-chemoradiotherapy setting due to the very high risk of breakdown.

### Primary Total Laryngectomy

Total laryngectomy remains the gold standard treatment for locally advanced T4 laryngeal cancers with gross cartilage destruction or extralaryngeal extension, as well as for treatment of locally recurrent laryngeal cancers after primary non-surgical treatment. The rationale for primary total laryngectomy in advanced T4 cases is the decreased likelihood of complete response with radiotherapy or chemoradiotherapy;^46^ the lack of evidence regarding non-surgical management of such cases, as large volume T4 cases were excluded from many of the organ preservation studies;^16^ the reduced success rate of salvage laryngectomy in the setting of extralaryngeal disease; and the increased incidence of major complications after salvage laryngectomy.^45^

In the past, primary total laryngectomy was also recommended in patients with bulky T3 tumors. With the advent of organ preservation protocols and evidence from the VA and RTOG studies, the number of total laryngectomies performed for T3 disease has reduced substantially. However, there is probably still an important role for primary total laryngectomy in selected patients with T3 primary tumors. An example of a case where primary total laryngectomy would be a very reasonable option is that of a young patient with good intelligence and social support, who has a T3 bulky transglottic SCC with fixed vocal cord fixation, a compromised airway, and questionable cartilage destruction on CT scan. The major arguments in favor of consideration of total laryngectomy in such a cases include adverse characteristics of primary tumor which may increase the risk of persistence or local recurrence, including large size,^47^ vocal cord fixation,^13^,^48^ and transglottic tumor extent; the presence of pre-treatment laryngeal dysfunction which portends a higher risk of permanent laryngeal dysfunction after even successful non-surgical treatment; and good patient performance status, intelligence, motivation, and social support which predicts a better likelihood of good speech and other functional outcomes after total laryngectomy.

Total laryngectomy is a major operation with significant functional, social, and psychological consequences for the patient. The major functional impact is due to loss of voice. The best method for speech rehabilitation would appear to be surgical voice restoration with tracheo-esophageal speech after tracheo-esophageal prosthesis placement.^49^ A high success rate for surgical voice restoration is reported by many authors;^50^–^52^ however, other studies which have endeavored to capture and follow up all patients undergoing total laryngectomy report the use of successful tracheo-esophageal speech in around half of patients.^49^ Of those who do not achieve successful tracheo-esophageal speech, some will achieve reasonable esophageal speech. Speech outcomes with use of electrolarynx are generally poor. Up to one quarter of all patients do not achieve intelligible speech at all.^49^ Other issues after total laryngectomy include the presence of a stoma in the neck, with attendant need to take precautions to avoid water getting in and keeping it clean; less effective coughing, and inability to perform a Valsalva maneuver during abdominal straining or lifting; and loss of sense of smell. Most patients undergoing primary laryngectomy without pharyngeal resection have satisfactory swallowing. Dysphagia is more common after salvage laryngectomy which is usually related to post-radiotherapy stricturing.

Total laryngectomy has been reported to be effective in 67%–81% of patients with T3 tumors,^53^–^55^ and 55% of patients with T4 tumors.^54^ Local recurrence may take the form of stomal or peristomal recurrence, which is believed to arise from metastatic paratracheal nodes, or pharyngeal/base of tongue/esophageal recurrence, which probably arises due to unrecognized submucosal extension or local lymphovascular invasion.^56^ Risk factors for local recurrence include transglottic or subglottic tumor extent,^54^ lymph node metastases,^54^–^56^ poor differentiation,^54^ lymphovascular invasion,^56^ preoperative tracheostomy,^55^,^56^ and positive resection margins.^56^

### Salvage Treatment

With the increasing role of non-surgical management in the treatment of advanced larynx cancer, total laryngectomy is increasingly becoming as a salvage treatment for cases which fail radiotherapy or chemoradiotherapy. Salvage laryngectomy is associated with an increased risk of major complications including pharyngocutaneous fistula,^45^ enlargement of the tracheo-esophageal puncture site,^57^ and dysphagia. Additional risk factors for these complications in the salvage setting include interval since radiotherapy^45^ and concomitant performance of bilateral neck dissection.^45^ In an effort to reduce the risk of these complications, several authors have advocated elective use of pectoralis major myogenous flaps, placed in onlay fashion, or free flaps interposed between the pharynx and skin/stoma.^58^ The use of a pectoralis major myogenous flap to bolster the pharyngeal repair has been reported by some authors to reduce the incidence of pharyngocutaneous fistula, and shorten time to healing in cases which do fistulize.^59^,^60^ On the other hand, other authors found no significant difference in the incidence of fistula between patients undergoing and not undergoing pectoralis major flap.^45^,^61^ However, these studies were all retrospective, so it is not possible to exclude bias due to cases considered at higher risk of fistula having undergone pectoralis major flap.

## TREATMENT OF THE NECK

### No neck

Supraglottic cancers have a marked propensity to give rise to nodal metastases, with an incidence of metastases detected by pathological examination in the N0 neck of 21%–30%.^62^,^63^ Metastases usually occur at levels II and III,^64^,^65^ but, in the setting of established disease at these levels, level IV may also be involved.^66^ Involvement of levels I and V are less frequent.^65^ Bilateral neck metastases are common owing to the frequent midline location of the primary tumor.^67^ Thus, all patients with supraglottic cancer, even with clinically N0 necks, should undergo elective neck treatment. This may take the form of elective neck dissection at the time of surgical treatment of the primary, or elective nodal irradiation of at-risk nodal groups postoperatively^68^,^69^ or concomitant with laryngeal irradiation in patients undergoing primary non-surgical treatment.^69^

Although the risk of nodal metastases in patients with glottic cancer and clinically N0 necks is much lower, elective treatment of the ipsilateral neck in patients with advanced (T3/4) glottic cancers is generally recommended. This will usually involve elective nodal irradiation for patients undergoing non-surgical treatment. For advanced glottic cancers, particularly those with subglottic extension, paratracheal nodes should also be treated, due to risk of metastatic spread to these nodes.^70^

For patients undergoing primary total laryngectomy, elective neck dissection may be performed, and pathological information obtained from this may help inform the radiation oncologist in determining postoperative treatment fields. An alternative approach which may be particularly suitable to frail patients is to not perform neck dissection, in order to expedite the operation and minimize the risk of complications, and allow postoperative radiotherapy to also treat at-risk nodes. On the other hand, elective neck dissection in these patients usually does not add an excessive amount of time to the operation and, if pathological findings are favorable, may allow the patient to avoid postoperative radiotherapy altogether.

### N+ neck

Patients with clinically evident nodal metastases who are undergoing primary laryngectomy should undergo simultaneous unilateral or bilateral neck dissection, as appropriate, for definitive treatment of their metastatic neck disease. This will be followed in most cases by postoperative radiotherapy. More controversial is the management of clinically evident cervical metastases in patients undergoing primary non-surgical treatment. Over the last number of years, the efficacy of primary chemoradiotherapy in the treatment of the clinically positive neck has been extensively studied. These studies have shown an excellent rate of complete response, ranging from 83%–87% for N1 disease,^71^,^72^ to 63%–66% for N2 disease,^72^,^73^ and 40%–43% for N3 disease.^72^,^73^ Patients who fail to achieve a complete response in the neck may be successfully treated by neck dissection 6–12 weeks after completion of treatment,^72^ whereas neck dissection appears unnecessary in patients achieving complete response as the risk of neck failure in such cases is very low.^73^,^74^ Isolated regional recurrence appears uncommon in laryngeal cancer, with local recurrence or combined local and regional recurrence being far more common.^56^,^71^ Thus, primary chemoradiotherapy for patients with advanced laryngeal cancer with metastatic neck disease, with post-treatment neck dissection reserved only for those patients with incomplete radiological response in the neck, has become standard treatment in most institutions.^74^

For patients with large-volume neck disease which may be considered less likely to respond to radiotherapy, an alternative option is up-front neck dissection, followed by radiotherapy or chemoradiotherapy for treatment of the primary tumor and adjuvant treatment to the neck.^74^,^75^ This option may be particularly useful in patients with small primary tumors and bulky metastatic neck disease, as it may obviate the need of intensification of radiotherapy treatment with chemotherapy, provided there are no major adverse histological features (positive margins or gross extranodal extension) in the neck dissection specimen. Another advantage is obviating the additional morbidity of post-treatment neck dissection.^35^ One disadvantage is that if patients require total laryngectomy with flap reconstruction in the future, obtaining suitable recipient vessels for anastomosis may be more problematic.

### Salvage Surgery

Clinically evident nodal metastases at the time of recurrence require surgical extirpation simultaneous with laryngectomy. The treatment of cases with local recurrence of laryngeal cancer but without clinically evident nodal metastases is more controversial. Traditionally, many authors have recommended elective dissection of the N0 neck, particularly with supraglottic cancers, in which case bilateral neck dissection was commonly required.^76^,^77^ However, the wider availability of better preoperative imaging has allowed other authors to challenge the need for elective neck dissection in the salvage setting, particularly among patients who were staged N0 before initial treatment.^78^,^79^ The reported incidence of positive nodes in patients undergoing elective neck dissection at the time of salvage laryngectomy ranges from 3% to 19%.^45^,^76^,^77^,^79^–^82^ Possible reasons for the wide range include differences in study inclusion criteria, and differences in preoperative imaging studies used to stage the neck at the time of recurrence. In our institution, we found an incidence of occult neck disease of 8% (5% of dissected heminecks) among patients with clinically rN0 necks which had been staged radiologically with preoperative CT scan.^78^ Bilateral neck dissection at the time of salvage laryngectomy has been reported to lead to a higher incidence of major complications including pharyngocutaneous fistula.^45^,^78^,^80^ Furthermore, elective neck dissection in this group does not appear to confer any survival benefit.^56^,^80^,^81^ Therefore avoidance of neck dissection if feasible may be beneficial by reducing the morbidity and risk of complications of salvage laryngectomy.

### Outcomes of Treatment

Five-year overall survival rates for patients with advanced larynx cancer range from 48% to 54%.^32^,^43^,^44^ For the most part, this does not appear to be affected by choice of treatment, with the increased local recurrence rate seen in non-surgically treated patients offset by the ability of many of these patients to be salvaged by total laryngectomy at the time of recurrence. This would appear to lead to equal overall survival between surgically and non-surgically treated patients, but a higher rate of larynx preservation in the non-surgical group.^14^,^43^,^44^

However, with the increasing shift toward non-surgical treatment strategies, there are worrying recent reports regarding a decreased survival for larynx cancer.^17^ It has been suggested that this may be linked to less aggressive surgical treatment of the larynx and/or neck. Hoffman et al. reported the decreased survival in larynx cancer to be paralleled by increasing use of non-surgical management with radiation alone or chemoradiation, and found non-surgical treatment to be associated with higher mortality than surgical treatment of T3N0 glottic cancer.^17^ Other studies have reported better survival in surgically treated patients; however, it is impossible to exclude bias in many of these studies. Another possibility is the increased long-term toxicity from concurrent chemoradiation protocols, and an apparent trend toward increased death rates due to non-primary cancer-related causes.^34^ Further research will be required in the coming years to elucidate the causes of this apparent decrease in larynx cancer survival, and/or better select patients for surgical versus non-surgical treatment.

## CONCLUSIONS

The management of advanced laryngeal cancer has evolved toward a predominance of non-surgical strategies, in an endeavor to avoid the sequelae of total laryngectomy. This has been facilitated by the development of modern chemoradiotherapy protocols with improved local control compared to radiotherapy alone. Ongoing challenges include development of strategies to reduce toxicity and adverse functional outcomes. Most very advanced (T4) laryngeal cancers are best treated with up-front total laryngectomy, due to the lower likelihood of response with non-surgical treatment. The role of total laryngectomy is increasingly as a salvage procedure for cases failing radiotherapy or chemoradiotherapy. Of increasing concern are reports of reduced survival among patients with laryngeal cancer, and speculation that this may be linked to recent changes in treatment paradigms.

## Abbreviations:

RTOG: Radiation Therapy Oncology Group
SCC: squamous cell carcinoma
VA: Veterans Administration.
